# Where did I come from? Where am I going? Functional differences in visual search fixation duration

**DOI:** 10.16910/jemr.10.1.5

**Published:** 2017-03-04

**Authors:** Harold H. Greene, James M. Brown

**Affiliations:** University of Detroit Mercy, Detroit, Michigan, USA; University of Georgia, Athens, Georgia, USA

**Keywords:** Saccade direction, Fixation duration, Saccade preparation, Preview benefit

## Abstract

Real time simulation of visual search behavior can occur only if the control of fixation durations is sufficiently understood. Visual search studies have typically confounded pre- and post-saccadic influences on fixation duration. In the present study, pre- and post-saccadic influences on fixation durations were compared by considering saccade direction. Novel use of a gaze-contingent moving obstructer paradigm also addressed relative contributions of both influences to total fixation duration. As a function of saccade direction, pre-saccadic fixation durations exhibited a different pattern from post-saccadic fixation durations. Post-saccadic fixations were also more strongly influenced by peripheral obstruction than pre-saccadic fixation durations. This suggests that post-saccadic influences may contribute more to fixation durations than pre-saccadic influences. Together, the results demonstrate that it is insufficient to model the control of visual search fixation durations without consideration of pre- and post-saccadic influences.

## Introduction

Greene, Brown and Dauphin ( 6 ) found that during visual search, fixation durations preceding upward eye movements were briefer than fixation durations preceding downward eye movements. This vertical visual field asymmetry in pre-saccadic fixation durations (PSFDs) is similar to the vertical asymmetry reported for saccadic reaction times (SRTs) where SRTs are faster towards upper visual field (UpVF) than lower visual field( LoVF) targets when observers are instructed to make a single saccade as quickly as possible from a fixation point to a peripheral target (e.g., Tzelepi, Laskaris, Amditis, & Kapoula, ( 19 ); see also Greene et al., ( 6 ) for a review). It should be noted that SRTs which are typically influenced by salient cues, are not equivalent to fixation durations, which are heavily influenced by various task demands and endogenous processing (e.g., Henderson, Brockemole, Castelhano, & Mack, ( 7 ).

The vertical visual field asymmetry findings of Greene et al. (2014) suggest that PSFDs and SRTs behave in a manner that is functionally similar with respect to saccade direction. Whereas PSFDs are fixation durations *before saccades are executed *in a given direction (i.e.,how long the eyes stay before moving in some direction), post saccadic fixation durations (PoSFDs) are fixation durations *after saccades land from being executed *in a given direction(i.e., how long the eyes stay depending on whence they came). Obviously, PSFDs and PoSFDs are not independent of each other in a multi-fixation visual search task. The same PoSFD after a saccade has been executed in a particular direction serves as the PSFD for the ensuing saccade in the upcoming direction. Despite the lack of independence between PSFDs and PoSFDs, the functional similarity between SRTs and PSFDs suggest that PSFDs may primarily reflect competition between (peripheral) saccade preparation and (foveal) fixation stabilization mechanisms, as has been suggested for SRTs (e.g., Munoz & Fecteau, ( 10 ). Fixation stabilization would have to include the interaction of low-level saccade preparation, and high-level cognitive processing mechanisms. A saccade is executed when the direction-sensitive saccade mechanisms overcome fixation stabilization, in a winner-take-all manner ( 10, 18 ). In contrast to PSFDs, PoSFDs may primarily reflect information accrual (i.e., high-level cognitive processing mechanisms). Justification for this assertion comes from text-reading and scene-viewing studies that have utilized gaze-contingent screen manipulations of peripheral information (see Rayner, ( 15 ) for a review). In these studies, words or target objects which were visible in peripheral vision are changed during, or after a saccade that brought them into foveal processing range. The change imposed on the previewed item before fixation typically leads to an increase in the after-saccade fixation duration. These kinds of findings indicate that PoSFDs are influenced by peripheral preview in text-reading, and scene-viewing. A limitation of the gaze-contingent approach utilized in the text-reading and scene-viewing studies above is that they allowed access only to experimenter-determined elements of interest (i.e., fixation duration on target word or target object). It has been assumed that the findings generalize to all elements in a display.

**Figure 1.**
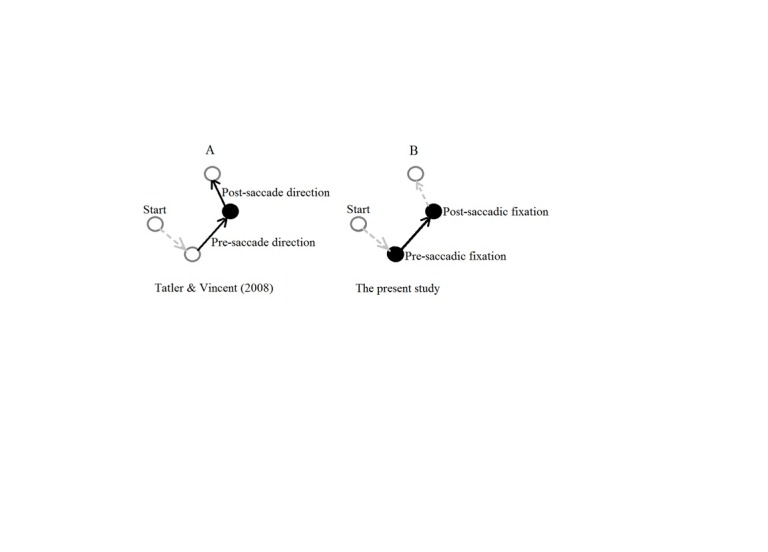
Schematic of a sequence of four fixations, denoted by circles, with arrows representing saccade direction between fixations. The solid black circles and solid arrows are of concern to the two studies shown. Panel A shows that Tatler and Vincent ( 17 ) were concerned about pre- and post-sacade changes in direction on fixation duration. Panel B shows that the concern of the present study was pre- and post-saccadic fixation durations with an intervening saccade.

Visual search may not recruit the same mechanisms as text reading, and scene viewing. During visual search, a simple rejection of a currently-fixated non-target may be sufficient for the eyes to be moved to a different point of interest. Compared to text reading and scene-viewing, during visual search, integration of information from a previous point of interest may be less necessary for successful completion of visual search. In effect, preview benefit may not be utilized as much during visual search. Previous visual search studies have not been designed to contrast PSFDs and PoSFDs. In fact, visual search studies conducted with or without gaze-contingent loss of peripheral information cannot compare PSFD and PoSFD, unless saccade direction information is considered. With concerns almost similar to the focus of the present study (i.e., PSFD and PoSFD with an intervening saccade), Tatler and Vincent ( 17 ) investigated *pre- and post-saccade direction *effects on fixation duration in a scene-viewing task. Figure 1 contrasts the concern of the present study with the concern of Tatler and Vincent ( 17 ). Tatler and Vincent ( 17 ) found that fixation durations were longer with greater angles of pre- to post-saccade direction change.

While their study is informative, it does not address PSFD/PoSFD issues. Without gaze-contingent manipulations, Tatler and Vincent’s ( 17 ) approach is also not amenable to quantifying peripheral preview effects in visual search (as has been done in text-reading and scene-viewing studies; see Rayner, ( 15 ). When gaze-contingent peripheral screen information manipulations have been utilized in visual search, researchers have *speculated* and attributed the general increase in fixation durations to a lack of peripheral benefit which led to higher PoSFDs ( 1, 5 ), or competition between fixation stabilizing and saccade preparation mechanisms, which led to higher PSFDs ( 2 ). By considering saccade directions, Greene et al. ( 6 ) revealed an asymmetry in PSFDs. However, the question of PoSFDs was not addressed. In effect, differences between PsFDs and PoSFD have not been well established for visual search. The current state of knowledge inspires two questions that are addressed in the present work. First, do PSFDs and PoSFDs reflect different *functional* mechanisms? Second, how do competition between saccadic mechanisms and peripheral preview rank in contributing to fixation durations?

*Question 1: Do PSFDs and PoSFDs reflect different functional mechanisms?*

While a vertical asymmetry is well-established for PSFDs, it is not known, however, how PoSFDs behave as a function of saccade direction. Given our earlier assertion that PoSFDs may primarily reflect information accrual (facilitated by preview benefit), it is reasonable to expect that PoSFDs may be longer after briefer PSFDs. Hence, PoSFDs may exhibit an asymmetry that is opposite to that of PSFDs. It is also possible that such an asymmetry may not be exhibited for visual search, given that preview benefit may not be much utilized during visual search. If PSFDs and PoSFDs demonstrate differences in asymmetry, this would suggest that they are functionally different in their moment-by-moment contributions to conscious vision. A functional difference would call into question, the popular practice of not distinguishing between the two in computational models of fixation duration (e.g. Nuthmann, Smith, Engbert, & Henderson, ( 12 ); Trukenbrod & Engbert, ( 18 ).

*Question 2: How do competition between saccadic mechanisms and peripheral preview rank in contributing to fixation durations?*

Eye movement visual search is driven primarily by endogenous shifts of attention (e.g., 7 ). As such, the durations of fixations during visual search reflect more than low-level competition between fixation stabilization and saccade preparation mechanisms. Given the assumption that PSFDs primarily reflect low-level competition between fixation stabilization and saccade preparation mechanisms (e.g., 10 ), and that PoSFDs primarily reflect the combination of pre- and post-saccade information accrual mechanisms (e.g., 15 ), it is important to determine relative contributions of both low-level, and information processing mechanisms to fixation durations. It is anticipated that knowledge of the relative contributions of both to fixation durations will contribute to constraining algorithms used to simulate realtime visual search behavior.

To this end, we have devised an approach based on the findings that a gaze-contingent change imposed on previewed items before a saccade, typically leads to an increase in the post-saccade fixation duration ( 5, 15 ). The approach involves quantifying the cost of obstructing one side of the peripheral visual field at a time, in a gaze-contingent manner. Given the difference found in UpVF and LoVF visual field processing during visual search ( 6 ), we blocked the UpVF and LoVF from view. It has been suggested that saccade preparation mechanisms are most active when saccades were directed to visible, locally-defined peripheral targets ( 3, 4 ). Hence, blocking the peripheral visual field should minimize competition from saccade preparation mechanisms, and should thus move the balance of competition towards fixation stabilization (and longer PSFDs). Blocking the peripheral visual field is also expected to minimize peripheral preview, leading to longer PoSFDs (see Rayner, ( 15 ) for a review). If only competition between fixation stabilization and saccade preparation mechanisms are responsible for increased fixation durations, then we expect PSFDs to be longer when ensuing saccades are directed towards the region that was blocked from view. As well, no effect of the visual field would be expected for PoSFDs. If however, only peripheral preview benefit mechanisms are responsible for increased fixation durations, then we expect PoSFDs to be longer if the preceding saccade was directed towards the obstructed visual field. No such increase in PSFDs is expected. Finally, if both types of mechanisms are involved (i.e., competition for dominance, and peripheral preview benefit), then we expect PSFDs and PoSFDs to be longer when saccades are directed towards the obstructed visual field. comparison of the effect sizes would revea which process (competition for dominance, or peripheral preview) is more influential in causing increased fixation duration during obstructed visual search. By inference, we would have quantified the ranking of competition between saccadic competition mechanisms and peripheral preview mechanisms, as they contribute to fixation durations.

## Methods

### Participants

Eighteen adults (18 - 54 years old; 5 male) at the University of Detroit Mercy participated in the experiment. All were naïve about the aim of the study, and all had normal or corrected-to-normal visual acuity. The experiment was part of a project approved by the Institutional Review Board at University of Detroit Mercy, and it was conducted in accordance with the Belmont Report, and the Code of Ethics of the World Medical Association (Declaration of Helsinki).

**Figure 2.**
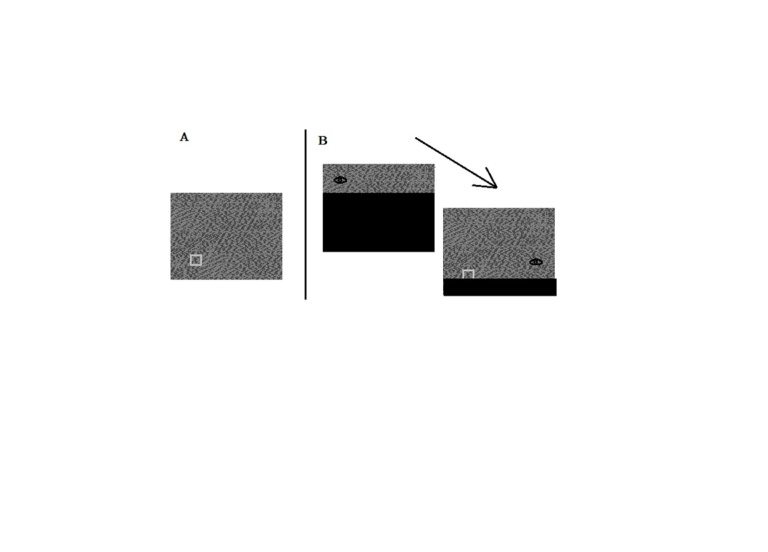
A. Sample stimulus display with a target square. The contrast of the target is exaggerated in the figure. B. Sample view of a participant who first looks to the upper left of the display (see the eye), and then to the lower right.

### Stimulus

The stimuli were multi-grey-level random dot noise images (e.g., Figure 2A). Images were 32 wide X 26 high. For each image, a target was embedded in one of 68 predefined locations along invisible concentric circles. The target was a low contrast square, 1.5 wide X 1.5 high.

### Apparatus

Random dot noise images were presented on a 17- inch monitor (60Hz refresh rate, 1024 X 768 pixels). Observers acknowledged finding the target by pressing the computer’s left mouse key. Eye positions were sampled at 500 Hz by an Eyelink II eye tracker that recorded saccades when eye velocity exceeded 30° s^−1^, or when eye acceleration exceeded 8000° s^−2^. The eye tracker was controlled by EYETRACK software ( http://blogs.umass.edu/eyelab/software/ ). Gaze-contingent obstructions in the peripheral fields were created using moving mask algorithms in EYETRACK. A peripheral obstruction was a black rectangular mask placed 2.5 above (UpVF obstruction) or 2.5 below (LovF obstruction) the current eye position on the random dot noise images. The size of the obstruction on the screen changed to hide the display, depending on the current eye position. For example, with a LoVF obstruction, an eye fixation at the upper left corner of a random dot noise image left most of the image hidden (Figure 2B). A Sper Scientific light meter 840006 was used to record ambient illumination in the laboratory.

### Procedure

Participants sat about 55 cm from the monitor in a welllit room (100 Lux). An experimental session started with a 9-point calibration of a headmounted Eyelink II eye tracker. Eye drift correction was performed before every search trial to maintain a high level of tracking accuracy. A trial consisted of a random dot noise stimulus (e.g., Figure 2A). Observers executed a saccadic search for the target in each stimulus, and terminated the trial with a mouse click response as soon as the target was localized. Each trial consisted of one of three viewing conditions: No Obstruction, Upper Visual Field Obstruction, and Lower Visual Field Obstruction. The obstruction (programmed as a gaze-contingent moving mask that hid everything below or above eye fixation) was located 2.5 degrees above, or below the fixation point. The obstruction followed the observers’ eye position with a delay that was brief enough not to disrupt a seamless perception of the visual stimuli. The target was presented one time at random in each of sixty-eight predefined target locations, for a total of 204 trials per observer (i.e., 68 target locations X 3 viewing conditions).

## Results and Discussion

**Figure 3.**
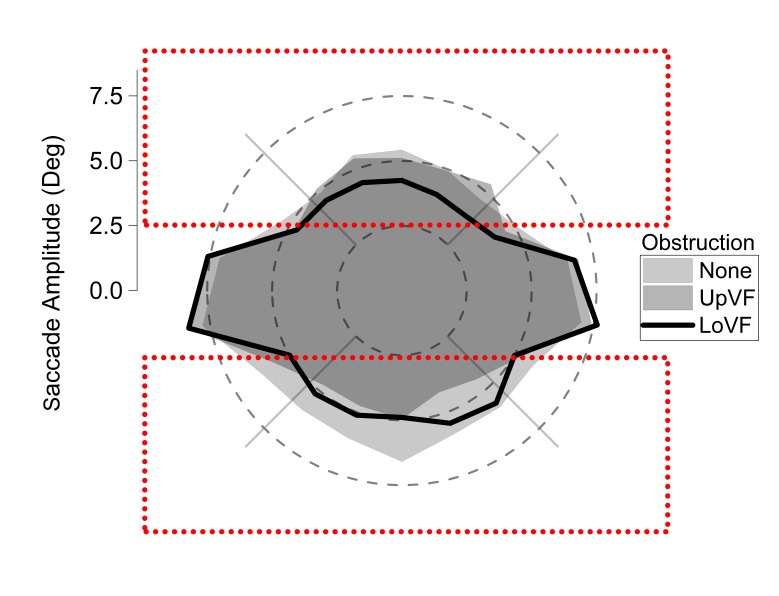
Saccade amplitudes as a function of saccade direction and obstruction condition. The rectangles show visual field obstruction locations in the upper or lower visual field, relative to eye fixation (i.e. 0 deg). Vertical saccades were executed beyond the 2.5 deg near boundaries of obstruction.

The hypotheses of the experiment depend on an influence of visual field obstruction, which was located 2.5 deg above or below eye fixation. As such, it was important to verify that saccades were made beyond the boundary of the obstruction, when it was present. One-sample t tests (comparing mean saccade amplitudes to a parameter of 2.5 deg) indicated that mean saccade amplitudes were significantly greater than 2.5 deg when there was no obstruction (all t(17) > 10.35; all ps < .001), in the Upper Visual Field Obstruction condition (all t(17) > 6.45; all ps < .001), and in the Lower Visual Field Obstruction condition (all t(17) > 4.95; all ps < .001). Indeed, saccades were typically made beyond the boundary of the obstruction. The polar plot in Figure 3 illustrates the amplitudes of saccades relative to upper and lower visual field obstruction.

**Figure 4.**
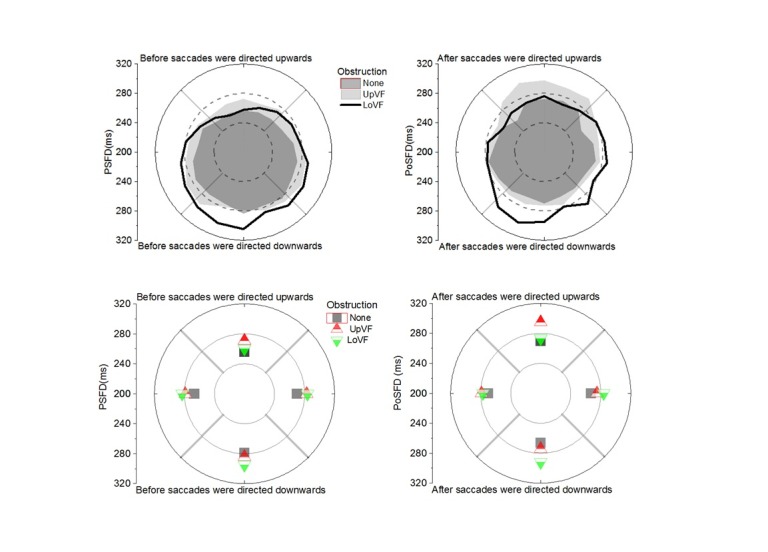
: (Left) Pre-Saccadic Fixation Durations and (Right) Post-Saccadic Fixation Durations as a function of saccade direction and obstruction condition. The panels on top depict fixation durations as a function of 20 deg saccade direction bins. The panels at the bottom depict fixation durations as a function of 90 deg saccade direction bins (i.e., Up, Down, Left, Right). All analyses in the text reflect a 3 Obstruction (None, Upper Visual Field Obstruction, and Lower Visual Field Obstruction) X 4 Saccade Direction (Up, Down, Left, Right) design.

The primary concern of the experiment was fixation durations made in search of the target. Following Greene ( 5 ), search fixation durations were defined as fixation durations made after the initial saccades but before the final fixations were terminated by key-press responses. Search fixation durations less than 90 ms and greater than 2000 ms were defined as outliers.

Across observers this criterion led to a removal of about 6% of search fixation durations. Each observer was left with between 1800 and 9600 fixation durations (median, 3775). These durations were then classified as PSFD, and PoSFDs, depending on saccade direction. As described earlier, PSFDs are fixation durations before the eyes go to a new location. PoSFDs are fixation durations after the eyes have come from some location in the display. Saccade directions were grouped in 90 deg bins (i.e., up, down, left, right) for statistical analysis. Two separate factorial ANOVAs—one for PSFDs and one for PoSFDs were conducted: 3 Peripheral Obstruction (None, Upper Visual Field Obstruction, and Lower Visual Field Obstruction) X 4 Saccade Direction (Up, Down, Left, Right).

### Major findings

Figure 4 shows that there was a Peripheral Obstruction X Saccade Direction interaction for PSFDs [F(6, 102) = 4.99, p < .01], and for PoSFDs [ F(6, 102) = 11.94, p < .01]. In effect, PSFDs before the eyes went off in a given direction, and PoSFDs after the eyes came from moving in that direction were influenced by obstruction in the visual field. As evident in Figure 4 and discussed below, the pattern of fixations durations as a function of saccade direction with and without an obstruction was different for PSFDs vs. PoSFDs.

### Minor findings

For PSFDs, there was a main effect of Peripheral Obstruction [F(2, 34) = 6.75, p <.01], and Saccade Direction [F(3, 51) = 6.75, p < .01]. While there was a main effect of Peripheral Obstruction [F(2, 34) = 6.87, p < .01] for PoSFDs, there was no effect of Saccade Direction [F(3, 51) = 0.78, p > .05].

*Question 1: Do PSFDs and PoSFDs reflect different mechanisms? *This question was addressed by analyzing asymmetries in PSFDs and PoSFDs in each of the three peripheral obstruction conditions of the experiment using Tukey tests. The main findings shown in Figure 4 are summarized in Table 1.

**Table 1:** PSFD and PoSFD Asymmetry (Asym) are not Equivalent as a Function of Gaze-Contingent Obstruction

Obstruction	PSFD	PoSFD
None	LoVF Asym	No Asym
UpVF	LoVF Asym	UpVF Asym
LoVF	LoVF Asym	LoVF Asym

1.*Asymmetry comparisons: Visual search with no peripheral obstruction.*

If PSFDs and PoSFDs reflect different mechanisms, it was reasonable to expect the vertical visual field asymmetry observed for PSFDs ( 6 ) would not be the same for PoSFDs. For this paragraph, the reader is directed to the dark region in the top left panel, or the squares in the bottom left panel of Figure 4. Analysis of the No Obstruction condition (similar to Greene et al., ( 14 ) showed that PSFDs were shorter by 23 ms for up-directed saccades (256ms) than down-directed saccades (279ms) [t(102) = 6.67, p <.01]. The LoVF-weighted vertical visual field asymmetry matches the asymmetry reported by Greene et al. ( 14 ). No significant difference in PSFDs was found for left-directed saccades (267ms) vs right-directed saccades (270ms), [t(102) = 0.99, p > .05] -- a finding also consistent with the results of Greene et al. ( 6 ). In sum, the vertical visual field asymmetry reported for PSFDs by Greene et al. ( 6 ) was replicated in the present study.

For this paragraph, the reader is directed to the dark region in the top right panel, or the squares in the bottom right panel of Figure 4. No significant difference was found for PoSFDs after up-directed saccades (270ms), compared to down-directed saccades (266ms) [t(102) = 0.99, p > .05]. Hence *in contrast to PSFDs, there was no vertical visual field asymmetry for PoSFDs*.There was also no horizontal asymmetry between left-directed saccades (270ms) and right-directed saccades (267ms) [t(102)= 0.99, p > .05]. In sum, when there was no obstruction in the visual field, PoSFDs did not behave the same way as PSFDs. This suggests that PSFDs and PoSFDs may reflect the operations of different mechanisms.

2.*Asymmetry comparisons: Visual search with UpVF peripheral obstruction.*

For this paragraph, the reader is directed to the light grey region in the top left panel, or the up-pointed triangles in the bottom left panel of Figure 4. PSFDs were shorter by 13 ms before up-directed saccades (271ms) than down-directed saccades (284ms) [t(102) = 3.92, p <.05]. This small LoVF-weighted vertical visual field asymmetry matches the asymmetry reported above, when there was no obstruction in the visual field. No significant difference in PSFDs was apparent before left-directed saccades (279ms) and right-directed saccades (283ms) [t(102) = 1.04, p > .05]. In sum, the results with a gaze-contingent obstruction in the UpVF are similar to those of Greene et al.( 6 ), who had no visual field obstruction.

For this paragraph, the reader is directed to the light grey region in the top right panel, or the up-pointed triangles in the bottom right panel of Figure 4. In contrast to PSFDs, PoSFDs were longer by 21 ms after up-directed saccades (295ms) than down-directed saccades (274ms) [t(102) = 5.76, p < .01]. This UpVF-weighted asymmetry was in the *opposite direction* of that reported above, for PSFDs. There was no significant difference in PoSFDs after left-directed saccades (279ms) vs right-directed saccades (275ms) [t(102)= 1.00, p > .05]. In sum, PoSFDs did not behave the same way as PSFDs, suggesting that they may reflect different mechanisms.

3.*Asymmetry comparisons: Visual search with LoVF peripheral obstruction.*

For this paragraph, the reader is directed to the black line in the top left panel, or the down-pointed triangles in the bottom left panel of Figure 4. PSFDs were shorter by 35 ms before up-directed saccades (260ms) than down-directed saccades (295ms) [t(102) = 10.49, p <.01]. The result reflects a LoVF-weighted vertical visual field asymmetry typical of PSFDs (see also Greene et al., 2014). No significant difference in PSFDs was found before left-directed saccades (283ms) vs right-directed saccades (284ms) [t(102) = 0.39, p > .05].

For paragraph, the reader is directed to the black line in the top right panel, or the down-pointed triangles in the bottom right panel of Figure 4. PoSFDs exhibited a LoVF-weighted asymmetry in that they were longer by 19 ms after down-directed saccades (292ms) than up-directed saccades (273ms) [t(102) = 5.33, p < .01]. This LoVF asymmetry was in the opposite direction of that found when a peripheral ob-struction was in the UpVF (see right panels in Figure 4). There was no significant horizontal asymmetry in PoSFDs (277ms vs 284ms after saccades were directed leftwards and rightwards, respectively) [t(102) = 1.83, p > .05]. In sum, PoSFDs did not behave the same way as PSFDs. Together, the asymmetry findings suggest that it is reasonable to conclude that PSFDs and PoSFDs reflect different mechanisms.

*Question 2: How do competition between saccadic mechanisms and peripheral preview rank in contributing to fixation durations?*

To address this question, the cost of peripheral obstruction to PSFDs and PoSFDs were analyzed. The logic was that there would be a greater cost of obstruction for the mechanisms that are more dominant in the adjustment of fixation durations. If competition between saccade and fixation mechanisms is more dominant than peripheral preview mechanisms, a greater cost of peripheral obstruction was expected for PSFDs than PoSFDs. In contrast, a greater cost was expected for PoSFDs than PSFDs if peripheral preview mechanisms are more dominant in the adjustment of fixation durations. The question was addressed by analyzing obstruction-induced increases in PSFDs and PoSFDs for saccades directed towards the gaze-contingent obstructions using Tukey tests.

1.*The cost of peripheral obstruction: Saccades directed upwards.*

For this paragraph, the reader is directed to the upper quadrants in the two left panels of Figure 4. With the UpVF obstruction, PSFDs for saccades directed upwards (271ms) were longer by 15 ms than PSFDs directed upwards when there was no obstruction (256ms) [t(102) = 4.31, p <.01, d= .58]. The LoVF obstruction had no effect on PSFDs for saccades directed upwards (260ms vs 256ms) [t(102)= 1.13, p > .05, d= .30]. Thus, *only the UpVF obstruction increased PSFDs for saccades directed upwards*.

For this paragraph, the reader is directed to the upper quadrants in the two right panels of Figure 4. With the UpVF obstruction, PoSFDs were longer by 25 ms after saccades were directed upwards (295ms) compared to PoSFDs directed upwards with no obstruction (270ms) [t(102) = 6.95, p <.01, d = .79]. The LoVF obstruction had no effect on PoSFDs for saccades directed upwards (273ms vs 270ms) [t(102) = 0.75, p > .05, d = .15]. Thus, *only the UpVF obstruction increased PoSFDs for saccades directed upwards*.

With respect to the question of cost, peripheral obstruction in the direction of up-directed saccades imposed a greater absolute cost for PoSFDs (25 ms) than for PSFDs (15 ms). As well, the related standardized effect size (Cohen’s d) was greater for PoSFDs than PSFDs (d= .79 vs d= .58). It is reasonable to conclude that peripheral preview mechanisms, not saccade-fixation competition mechanisms, were more dominant in the adjustment of fixation durations for saccades directed upwards.

2. *The cost of peripheral obstruction: Saccades directed downwards.*

For this paragraph, the reader is directed to the lower quadrants in the two left panels of Figure 4. With the LoVF obstruction PSFDs before saccades directed downward (295ms) were longer by 16ms than PSFDs directed downward with no obstruction (279ms) [t(102) = 4.95, p < .01, d= .80]. The UpVF obstruction had no significant effect on PSFDs (284ms vs 279ms) [t(102) = 1.56, p > .05, d = .23]. In sum, *only the LoVF obstruction increased PSFDs for saccades directed downwards.*

For this paragraph, the reader is directed to the lower quadrants in the two right panels of Figure 4. While the UpVF obstruction had no significant effect on PoSFDs after downward directed saccades (274ms vs 266ms) [t(102) = 2.18, p > .05, d= .32], the LoVF obstruction was associated with a 25 ms increase in PoSFDs (292ms vs 266ms) ([t(102) = 7.07, p < .01, d= 1.04]. In sum, *only the LoVF obstruction increased PoSFDs for saccades directed downwards.*

Cost-wise, peripheral obstruction in the direction of down-directed saccades had a greater absolute cost on PoSFDs than on PSFDs (25ms vs 17ms). Also, the related standardized effect size (Cohen’s d) was greater for PoSFDs than PSFDs (d= 1.04 vs d= .80). Together, the results suggest peripheral preview mechanisms, not saccade-fixation mechanisms, were more dominant in the adjustment of fixation durations for saccades directed downwards.

## General Discussion

The concern of the present study was the nature of PSFDs and PoSFDs during visual search. PSFDs were defined as fixation durations before the eyes moved in a given direction, and PoSFDs were defined as fixation durations after the eyes landed from the given direction. Given their similarity to SRT patterns, PSFD patterns were assumed to be primarily reflective of low-level competition between saccadic and fixation stability mechanisms, such that a saccade is executed when saccade preparation mechanisms overcome fixation stabilization ( 10 ). Hence, selective minimization of competition from saccade preparation mechanisms was expected to increase selectively, PSFDs. In contrast, PoSFD patterns (which have not been specifically addressed in visual search) were assumed to be reflective of the interaction of pre- and post-saccade information accrual. Selective prevention of preview was expected to increase selectively, PoSFDs.

Novel use of a gaze-contingent moving obstructer paradigm addressed two open questions related to visual search. The first question dealt with functional similarity between PSFDs and PoSFDs*.* If PSFDs and PoSFDs reflect different mechanisms, it was expected that saccade direction would differentially influence PSFD and PoSFD patterns. Results showed that PSFD patterns exhibited a vertical visual field asymmetry that was weighted towards the LoVF, irrespective of the location of a peripheral obstructer. This was similar to PSFD patterns reported by Greene et al. ( 6 ). However, PoSFD patterns exhibited this LoVF-weighted asymmetry only when the obstructer was in the LoVF.

These findings (summarized in Table 1) support the argument that PSFDs and PoSFDs reflect functionally different mechanisms. The second question of interest was the relative contribution of PSFDs and PoSFDs to visual search fixation duration. Although an obstructer in the UpVF always selectively increased PSFDs and PoSFDs, the increase (i.e., cost) was greater for PoSFDs. The same was true in the opposite direction for an obstructer in the LoVF. Thus, the findings suggest that PoSFDs (which have been assumed to reflect primarily the interaction of pre- and post-saccade information accrual) contribute more to the duration of fixations than PSFDs (which were assumed to reflect saccade competition mechanisms). Together, the results of the present study suggest that it is insufficient to theorize about the control of fixation durations without consideration of differential influences on PSFDs and PoSFDs.

Computational simulations facilitate understanding of biological mechanisms. Many computational models of looking behavior have focused on where saccades are directed (e.g., ( 8, 11, 13, 14, 20 ). Real time simulation of looking behavior can occur only if the control of fixation durations is sufficiently understood. The most relevant model with respect to visual search fixation duration is Trukenbrod & Engbert’s ( 18 ) model (ICAT). ICAT assumes that fixation durations are adjusted both directly (by currently-fixated information) and indirectly (by prior experience with processing demands). While the model accounts for global shifts in fixation duration from processing demands (i.e., indirect influences), and local variations in fixation duration (i.e., direct display influences), simulated distributions of fixations do not take into account PSFD patterns (e.g., ( 6 ) and differences between PSFD and PoSFD patterns, as demonstrated in the present study.

Interestingly, the PSFD asymmetry reported here (and by Greene et al. ( 6) ) is the opposite of the asymmetry observed when observers are instructed to respond manually to the onset of a target in the upper or lower visual field. Manual reaction times (MRTs) tend to be faster for targets in the *lower,* not upper visual field ( ( 9 ); see also Skrandeis, ( 16 ) for a review). In the present context, MRTs quantify a shift of attention when a target becomes visible. Tzelepi et al., ( 19 ) have speculated on the reason for the dissimilarity in SRT and MRT patterns. In their study, observers were asked either to attend covertly, or make a saccade to stimuli presented in the visual field. As expected, SRTs were faster for saccades towards UpVF than LoVF targets. Beyond this typical finding, magnetoencephalography (MEG) results indicated that frontal lobe areas involved in saccade preparation were accessed earlier and with less cortical activation for saccades about to be directed into the UpVF, than for the saccades about to be directed into the LoVF. The temporal advantage and greater automaticity in frontal processing may both contribute to faster SRTs into the UpVF. In contrast, when observers were instructed to attend covertly (without eye movements), dorsal cortex regions were activated earlier than ventral regions, and cortical activation was lower if attention was directed to LoVF compared to UpVF. *This* temporal advantage and greater automaticity in response to LoVF targets may contribute to faster MRTs for targets in the LoVF.

### Conclusion

The findings of the present study demonstrate that fixation duration control is differentially influenced by whence a saccade was directed (i.e. PoSFD control) and whither a saccade is to be directed (i.e., PSFD control). The results suggest a larger influence of preview (when a fixation lands after a saccade) than item selection (before a saccade is initiated). Of course, generalization of the findings may depend on the properties of the visual task (e.g. visual search vs passive scene viewing). The takehome message from the present findings is that fixation durations are influenced differently by pre-saccadic and post saccadic mechanisms, as a function of saccade direction. Future computational models of fixation duration control should consider pre- and post-saccadic influences as a function of saccade direction.

## Acknowledgements

Contributions made by research assistants (Ellen Day, Vanessa Osantoski) and by formal and informal reviewers of this work are appreciated. The authors declare that there is no conflict of interest regarding the publication of this paper.

## References

[b1] Bertera J. H., & Rayner K. (2000). Eye movements and the span of the effective stimulus in visual search. Perception & Psychophysics, 62(3), 576–585. 10.3758/BF03212109 0031-5117 10909248

[b2] Cornelissen F. W., Bruin K. J., & Kooijman A. C. (2005). The influence of artificial scotomas on eye movements during visual search. Optometry and Vision Science, 82(1), 27–35.1040-5488 15630401

[b3] Edelman J. A., & Goldberg M. E. (2001). Dependence of saccade-related activity in the primate superior colliculus on visual target presence. Journal of Neurophysiology, 86(2), 676–691. 10.1152/jn.2001.86.2.676 0022-3077 11495942

[b4] Edelman J. A., & Goldberg M. E. (2003). Saccade-related activity in the primate superior colliculus depends on the presence of local landmarks at the saccade endpoint. Journal of Neurophysiology, 90(3), 1728–1736. 10.1152/jn.00016.2003 0022-3077 12736233

[b5] Greene H. H. (2006). The control of fixation duration in visual search. Perception, 35(3), 303–315. 10.1068/p5329 0301-0066 16619948

[b6] Greene H. H., Brown J. M., & Dauphin B. (2014). When do you look where you look? A visual field asymmetry. Vision Research, 102, 33–40. 10.1016/j.visres.2014.07.012 0042-6989 25094053

[b7] Henderson J. M., Brockmole J. R., Castelhano M. S., & Mack M. (2007). Visual sali-ency does not account for eye move-ments during visual search in real-world scenes. Eye movements: A window on mind and brain (pp. 537–562). Amsterdam: Elsevier.

[b8] Itti L., & Koch C. (2000). A saliency-based search mechanism for overt and covert shifts of visual attention. Vision Research, 40(10-12), 1489–1506. 10.1016/S0042-6989(99)00163-7 0042-6989 10788654

[b9] Maehara G., Okubo M., & Michimata C. (2004). Effects of background color on detecting spot stimuli in the upper and lower visual fields. Brain and Cognition, 55(3), 558–563. 10.1016/j.bandc.2004.04.003 0278-2626 15223202

[b10] Munoz D. P., & Fecteau J. H. (2002). Vying for dominance: Dynamic interactions control visual fixation and saccadic initiation in the superior colliculus. Progress in Brain Research, 140, 3–19. 10.1016/S0079-6123(02)40039-8 0079-6123 12508579

[b11] Najemnik J., & Geisler W. S. (2009). Simple summation rule for optimal fixation selection in visual search. Vision Research, 49(10), 1286–1294. 10.1016/j.visres.2008.12.005 0042-6989 19138697

[b12] Nuthmann A., Smith T. J., Engbert R., & Henderson J. M. (2010). CRISP: A computational model of fixation durations in scene viewing. Psychological Review, 117(2), 382–405. 10.1037/a0018924 0033-295X 20438231

[b13] Parkhurst D., Law K., & Niebur E. (2002). Modeling the role of salience in the allocation of overt visual attention. Vision Research, 42(1), 107–123. 10.1016/S0042-6989(01)00250-4 0042-6989 11804636

[b14] Rao R. P., Zelinsky G. J., Hayhoe M. M., & Ballard D. H. (2002). Eye movements in iconic visual search. Vision Research, 42(11), 1447–1463. 10.1016/S0042-6989(02)00040-8 0042-6989 12044751

[b15] Rayner K. (2009). Eye movements and attention in reading, scene perception, and visual search. Quarterly Journal of Experimental Psychology, 62(8), 1457–1506. 10.1080/17470210902816461 1747-0218 19449261

[b16] Skrandies W. (1987). The upper and lower vis- ual field of man: electro-physiological and functional differences. Process in Sensory Physiology, 1-93.

[b17] Tatler B. W., & Vincent B. T. (2008). System- atic tendencies in scene viewing. Journal of Eye Movement Research, 2(2), 1–18.1995-8692

[b18] Trukenbrod H. A., & Engbert R. (2014). ICAT: A computational model for the adaptive control of fixation durations. Psychonomic Bulletin & Review, 21(4), 907–934. 10.3758/s13423-013-0575-0 1069-9384 24470305

[b19] Tzelepi A., Laskaris N., Amditis A., & Kapoula Z. (2010). Cortical activity preceding vertical saccades: A MEG study. Brain Research, 1321, 105–116. 10.1016/j.brainres.2010.01.002 0006-8993 20079341

[b20] Zelinsky G. J. (2008). A theory of eye movements during target acquisition. Psychological Review, 115(4), 787–835. 10.1037/a0013118 0033-295X 18954205 PMC2577318

